# Trust, Respect, and Reciprocity: Informing Culturally Appropriate Data-Sharing Practice in Vietnam

**DOI:** 10.1177/1556264615592387

**Published:** 2015-07

**Authors:** Laura Merson, Tran Viet Phong, Le Nguyen Thanh Nhan, Nguyen Thanh Dung, Ta Thi Dieu Ngan, Nguyen Van Kinh, Michael Parker, Susan Bull

**Affiliations:** 1 Oxford University Clinical Research Unit, Ho Chi Minh City, Vietnam; 2 Children’s Hospital 1, Ho Chi Minh City, Vietnam; 3 Hospital for Tropical Diseases, Ho Chi Minh City, Vietnam; 4 National Hospital for Tropical Diseases, Ha Noi, Vietnam; 5 University of Oxford, UK

**Keywords:** data sharing, clinical data, ethics, biomedical data, Vietnam, qualitative methods, information dissemination, access to information

## Abstract

International science funders and publishers are driving a growing trend in data sharing. There is mounting pressure on researchers in low- and middle-income settings to conform to new sharing policies, despite minimal empirically grounded accounts of the ethical challenges of implementing the policies in these settings. This study used in-depth interviews and focus group discussions with 48 stakeholders in Vietnam to explore the experiences, attitudes, and expectations that inform ethical and effective approaches to sharing clinical research data. Distinct views on the role of trust, respect, and reciprocity were among those that emerged to inform culturally appropriate best practices. We conclude by discussing the challenges that authors of data-sharing policies should consider in this unique context.

The sharing of individual-level clinical research data is increasingly seen as a high priority by researchers, medical publishers and research funders in high-income settings. Data sharing offers clear potential to expedite scientific innovation, increase the efficiency of research investment and improve the quality of analysis (Mello, Francer, & Wilenzick, 2013). In response to these potential benefits, an emerging consensus around the development of policy, infrastructure, and best practice in data sharing has arisen. To date, these activities have been concentrated in high-income regions where well-resourced institutions explore options for sharing their large volumes of research outputs. Initiatives in these settings have resulted in a variety of solutions to the technical, governance, practical, and ethical challenges of this new paradigm in research enhancement (Gøtzsche, 2011). There are, however, fewer initiatives to inform the development of solutions for the same challenges in low- and middle-income settings (Manju & Buckley, 2012).

This article focuses on initiatives where clinical data generated in biomedical research are made available to secondary researchers to address novel research questions without direct collaboration with the primary researchers. The roots of this movement in clinical research stem from the Human Genome project, and founding models for data release were set out in the Bermuda Principles and the Fort Lauderdale Agreement (Human Genome Organisation, 1996, 1997; Wellcome Trust, 2003). Data sharing has gained momentum in recent years as an increasing number of stakeholders have embraced the potential benefits and formulated policies that promote and in some cases mandate data sharing (European Federation of Pharmaceutical Industries and Associations & The Pharmaceutical Research and Manufacturers of America, 2014; Godlee & Groves, 2012; National Institutes of Health, 2015; Nature Journal, 2015; Public Library of Science, 2015; Science Magazine, 2015; Walport & Brest, 2011; Wellcome Trust, 2010).

The vast majority of these policies originate from institutions in high-income countries. There is, however, an increasing recognition of the importance of medical research conducted in low- and middle-income settings to address the disproportionate disease burden faced by these countries (The Interacademy Medical Panel, 2013). This has driven recent growth in both the volume and diversity of global health research funding, collaborations, studies, and outputs. The terms of these initiatives increasingly include requirements to share the individual-level data collected. This has created an urgent need for research to inform the development of effective, ethical, and sustainable approaches to data sharing in low- and middle-income settings. To address the current dearth of evidence in this area, the Public Health Research Data Forum funded an international collaborative study comprising a systematic literature review (Bull, Roberts, & Parker, 2015) and empirical studies in five low- and middle-income countries with strong local and international research programs (Cheah et al., 2015; Denny, Silaigwana, Wassenaar, Bull, & Parker, 2015; Hate et al., 2015; Jao et al., 2015; Parker & Bull, 2015). The aim was to explore the data-sharing experiences, attitudes, perceptions, and expectations of stakeholders in health research, to inform the development of best practices in each region. In Vietnam, the Oxford University Clinical Research Unit, the Hospital for Tropical Diseases, the Children’s Hospital 1, and the National Hospital for Tropical Diseases implemented the study. The Oxford University Clinical Research Unit has held strong research collaborations with each of these hospitals for up to 24 years (Wertheim, Barton, Merson & Thwaites, 2014). These collaborations have produced hundreds of clinical, laboratory, and epidemiological studies and associated datasets. The dynamics of these collaborations serve as a microcosm of the developing clinical research landscape in Vietnam (Sahoo, 2012).

With increasing research outputs, driven both locally and through international collaborations, the Vietnamese research governance framework is rapidly evolving to accommodate the increased scope and volume of activity. Sharing of individual-level clinical research data is not specifically addressed in existing research regulations or guidelines to date. There are, however, a number of recent regulations that may influence the development of guidelines on the type of information that can be shared, with whom it can be shared, and what can be done with it (see Box 1).

**Box 1.** Existing Vietnamese Regulations, Which Should Be Considered When Developing Data-Sharing Guidelines.

• Ordinance on the State of Emergency in Case of Great Disasters or Dangerous Epidemics, Vietnam Government (2002)
• Decision on Protection of Confidential Information in the Government Health Care Sector, Vietnam Ministry of Health (2005)
• Law on Intellectual Property Rights, Vietnam Government (2005)
• Law on Prevention and Control of Infectious Disease, Vietnam Government (2007)
• Regulation on Information and Reports on Epidemic Contagious Diseases, Vietnam Ministry of Health (2010)
• Circular on Guiding the Confidential Protection of Trial Data in Drug Registration, Vietnam Ministry of Health (2010)
• Official Instruction for the Management of Scientific Research, Ho Chi Minh City Department of Health (2011)
• Regulation on Speaking and Providing Information to the Press, Vietnam Ministry of Health (2013)

None of the existing Vietnamese laws or guidelines conflict with the principles or activities of data sharing, with the exception of restrictions on initial reporting of severe infectious diseases. The Official Instruction for Management of Scientific Research describes how to manage research activities including the sharing of research results as abstracts (Ho Chi Minh City Department of Health, 2011). The Circular on Guiding the Confidential Protection of Trial Data in Drug Registration details how to deal with confidential data in clinical trial results submitted for registration purposes (Vietnam Ministry of Health, 2010). These two documents most closely relate to how research outputs can be shared. The gap between the topics addressed in these documents and current international models of non-collaborative data sharing need to be addressed through local research, engagement, and policy development. Addressing this gap is central to the objectives of this study.

Although non-collaborative sharing of clinical research data is relatively novel in Vietnam, successful examples of sharing public health data are available. The Ha Noi School of Public Health (2014), Vietnam’s foremost public health institution, for example, has clear guidelines to govern the use of shared datasets for secondary analysis by students. Demographic surveillance studies such as the Survey and Assessment of Vietnamese Youth (SAVY) offer a successful example of data sharing by the provision of open online access to survey data from 9,000 households across the country (Vietnam Ministry of Health & General Statistics Office, 2003).

The ethical importance of exploiting the benefits of data sharing is especially critical to Vietnam. Maximizing the scientific benefits of research to improve patient outcomes locally will help to address current global inequalities in research resources and disease burden. Furthermore, meeting the data-sharing requirements of international funders and publishers is imperative to avoid compromising access to their support. But international data-sharing policies should not be imposed without consideration of local research culture, needs, and expectations. This study explores stakeholders’ understandings, perceptions, experiences, attitudes, and concerns about sharing individual-level clinical data. This article investigates expectations of what good Vietnamese data-sharing practice should be. We also address how to support the development of a culturally appropriate data-sharing practice and locally acceptable policy inVietnam .

## Method

This study is a qualitative assessment of views on data sharing of health research stakeholders in Vietnam. A consortium of investigators designed this study collaboratively for implementation across five sites (Parker & Bull, 2015). The central design was adapted in Vietnam to collect the most locally relevant information. Data collection took place across Vietnam between March and October 2014.

A total of 48 participants were selected by purposive sampling with a focus on maximizing diversity of background, experience, role, and geography (Table 1). Participants included government officers (2) with roles in research policy development; ethics committee members (7), with research experience and a role in decision making at a major Vietnamese research institution; researchers (24) from a cross-section of educational backgrounds and experiences, working in local and international, academic and commercial institutions; and clinical research participants enrolled in observational, interventional or cohort studies, with their family members (15) from northern and southern, urban and rural centers.

**Table 1.** Participant Composition by Data Collection Method.

	Male	Government employed	Vietnamese national	Urban dweller
Personal interviews	17/28 (60%)	18/28 (64%)	24/28 (86%)	28/28 (100%)
Focus group discussions	8/20 (40%)	0/20 (0%)	17/20 (85%)	14/20 (70%)
All participants	25/48 (52%)	18/48 (38%)	41/48 (85%)	42/48 (88%)
Refusals	1/2 (50%)	2/2 (100%)	2/2 (100%)	2/2 (100%)

Individual interviews were selected as the preferred data collection method for most researchers, government officers, and ethics committee members to create a secure environment for the open sharing of ideas. Focus group discussions were used to exchange ideas between those less familiar with data sharing, including junior researchers, research participants, and the participant’s family members. Interviews and discussions were recorded, transcribed verbatim, and checked against the recordings, and then translated to English (where required), de-identified, verified, and uploaded to NVivo 10 software.

In-depth interviews were guided by open-ended questions based on an interview guide (available as Supplementary Material to the online edition of this article at jre.sagepub.com/supplemental) developed iteratively throughout the study in discussion with the other research sites. The interview questions addressed knowledge, experiences, attitudes, concerns, expectations, governance, and culturally appropriate means of data sharing. Questions were selected on the basis of the participant’s experiences and responses as well as based on the need for further exploration of particular topics.

Three focus group discussions with research participants and their family members (collectively referred to as patient representatives) were facilitated. Research participants were selected based on recent or current enrollment into one of three studies led by a Vietnamese doctor at a Vietnamese government hospital: a longitudinal influenza surveillance study (rural northern Vietnam), an observational study of dengue infection (urban northern Vietnam), or a clinical trial of tuberculosis meningitis treatment (urban southern Vietnam). An additional focus group discussion was also organized with junior researchers in Ho Chi Minh City. Discussion topics and questions were piloted before recruitment to tailor context-specific language, optimize structure, and ensure that questions were appropriately focused and/or open. The choice to collect data via discussion group was made to facilitate the exchange of opinions among group members who had less familiarity with data sharing. Discussions began with an introductory session on terms, definitions, and examples of data, data management, and models of data analysis to inform the discussion. Study staff endeavoured to provide information about data sharing in an unbiased way, drawing on discussions in the literature about perceived advantages and concerns. Researchers were selected via snowball sampling after the initial identification of individuals interested in the topic and those whose work was subject to data-sharing requirements.

The validity of data collected was enhanced through triangulation as questions overlapped participant groups and data collection methods to approach the same topics in a variety of ways. Topics were discussed with diverse stakeholders to validate responses through multiple sources and allow for a range of opinions on the same topics. Responses from interviews and group discussions were consolidated and compared for analysis. Preliminary findings were discussed via fortnightly teleconferences with collaborators at the five project sites. Data were initially coded with descriptive nodes generated from an analytic framework developed during a collaborative workshop (Smith & Firth, 2011). The initial coding structure was then expanded to include inductive descriptive codes generated through close reading of the Vietnamese data (Thomas, 2006). Data were initially coded by T.V.P. in collaboration with L.M. The coding framework was refined following the cross-coding of a sample of transcripts by investigators from the five study sites. During a cross-site analysis meeting, themes emerging from the Vietnamese data were charted and discussed with co-investigators.

### Ethical Considerations

This study, including the protocol, informed consent forms, and outline interview guides were reviewed and approved by the ethics or institutional committees of the Hospital for Tropical Diseases, Ho Chi Minh City, Vietnam; Children’s Hospital 1, Ho Chi Minh City, Vietnam; National Hospital for Tropical Diseases, Ha Noi, Vietnam; Provincial Center for Preventive Health Care, Ha Nam, Vietnam; and Oxford Tropical Research Ethics Committee, Oxford, United Kingdom (OXTREC, Reference 1051-13).

Before initiating the study, all interview and group discussion participants had the study explained to them in a way that allowed them to understand the purpose, procedures, risks, benefits, and alternatives to study participation. Those who agreed to take part were asked to sign a consent form to confirm that they had been adequately informed about the study and that they agreed to participate. All participants were compensated with a small amount of cash (<US$10) or provided with lunch for the time spent participating in the study. Participants who traveled to the study site were also reimbursed an amount based on the distance traveled according to standard policy (average amount ~US$10). Study tools are available in the online appendix (http://jre.sagepub.com/supplemental) of this article. Interview transcripts are available, please contact the corresponding author for details. Information that could identify participants have been redacted to preserve privacy.

## Results

### Views About a Novel Initiative

There was interest among all study participants in how data can be shared, though very few had ever practiced non-collaborative data sharing or had any prior awareness of the potential issues that could arise. Knowledge of the increasing requirements for data sharing by journals and funders was also uncommon. Among the researchers interviewed, collaborative data sharing and cross-institutional partnerships were common with national and international collaborators, as well as with students for educational purposes.

The current dearth of non-collaborative data sharing among Vietnamese researchers was not perceived as a problem. The novel concept of sharing was introduced to study participants as an initiative from international funders, scientific publishers, and institutions. There was general acceptance that this would intuitively fit into the framework of Vietnamese research as a part of ongoing development through education, grants and publication involving international partners. This view was summed up by researchers as follows:

I think it [data-sharing practice] is the same everywhere in the world. Science is not the invention of Vietnamese people. What and how we do science, we learn from teachers. (Researcher–Male-I-18)

It [data-sharing practice] is the same everywhere. You don’t go to your neighbors to explain everything about what you’re doing. For me, the problem is not active refusal to give information. I don’t think it is an active process: people trying to keep information. Just for everybody, probably all humans, it’s not natural to give out all information all the time. So I don’t think it is a problem just in Vietnam because I travel a lot: Africa, Europe, US . . . I don’t think it is worse here. It is not different, not better. (Researcher–Male-I-26)

Despite acceptance that data sharing may become more common, it was not seen as a current priority. Multiple researchers referred to an “unwritten rule” that requires researchers to focus on the scientific and technical aspects of a project in development, while minimizing the paper work. An ethics committee member clarified this by explaining,

. . . no one can imagine the way ahead because they only pay attention to the technical aspects [of research]. Data sharing is not a priority [in Vietnam] at this point. (Ethics committee member–Male-I-15)

This was supported by a number of researchers who said that the need to promote or develop policy on data sharing was not a top priority in the competitive and resource-limited realm of academic science.

When patient representatives were first asked if they were willing to share their data, there was some reluctance. In each case, as information was provided about the type of data that could be shared and for what purpose, this quickly evolved to join the collective acceptance of this new initiative with a prevailing attitude of benevolence and trust:

You know, when I was invited here, I just thought it’s because I’ve been recruited to research on dengue fever in this hospital. . . . One staff informed me something about the possibility of sharing of my personal information . . . and my initial reaction was objection . . . then from the beginning of this discussion, I changed my mind and I am now willing to share information. It’s a change between acceptable and unacceptable. Now that I know sharing my information is for common good sake, I have changed my mind, and I am willing to allow sharing. But ideally, everything should be anonymized. (Patient representative–Male-G-02-03)

Vietnam is now in a transition period. We must sacrifice and accept inconvenience for development and we are strongly willing to contribute. Information is such a trivial thing to consider. Moreover sharing of my information doesn’t affect my life in any way. (Patient representative–Female-G-01-07)

### Principle Versus Practice

Although the general principle of data sharing as a growing global trend with an upcoming role in Vietnamese research was accepted, a different picture emerged when researchers were asked about sharing data from their own studies. During initial discussions, researchers often disagreed with the notion that sharing their individual-level data was acceptable. One ethics committee member embodied the typical response heard from many others by claiming,

Requesting raw data from someone? It is synonymous with being disrespectful! (Ethics committee member–Female-I-09)

Fueled by the intention of maintaining patient privacy and academic competitive advantage, clinical research data have long been protected as confidential information. Promoted by the confidentiality that was historically inherent to pharmaceutical industry trials, this mind-set is also prevalent in Vietnam. This may have contributed to the frequent (and often strong) initial objections to sharing the data from one’s own research. The idea that research data belong to the funder and that such ownership precludes the investigator from sharing was also a commonly quoted barrier. The public nature of sharing data directly challenged Vietnamese researchers’ views on data confidentiality. When considering the implementation of non-collaborative data sharing, researchers and ethics committee members made it clear that discussions of any data-sharing plans should occur at the initial stage of a research project.

### Views About Acceptable Data Sharing

After further discussion of some of the benefits and risks of sharing data, the participating researchers were asked to consider under what conditions they would or would not agree to share their data. As most researchers interviewed for this study were not yet engaged in non-collaborative data sharing, a theoretical exchange of ideas ensued on what the researchers envisaged as acceptable data sharing. There was consensus that secondary uses of data must contribute to scientific knowledge. This concept was central to most discussions, with particular emphasis placed on data uses that addressed the health needs and priorities of the Vietnamese community.

Next on the list of requirements for acceptability were criteria that demonstrate respect to the data provider, including transparent exchange of information, timely analysis, and feedback to the data provider:

. . . there is a condition: it must be fair. If you receive my data, you should give me feedback, I mean everything I need as part of a collaborative relationship. If sharing is unfair, no one wants to do anything. (Ethics committee member–Male-I-15)

Avoidance of conflict with the outcomes of the primary research was also considered important:

For a student, I think it is acceptable to share data to give them the direction for their thesis. For a colleague, if their purpose of study is different from mine, for example, I do clinical research but my colleague only requests epidemiological data which does not interfere with my final results . . . then I think it is acceptable. A pharmaceutical company might wish to use my data as control group, which is acceptable if their research doesn’t affect my final results . . . if their research design is similar to mine, then it interferes. (Ethics committee member–Female-I-22)

Researchers also discussed the importance of respecting the ethical commitments made when the data were collected:

I think the most important point is who ‘stands in front of the patients’! I mean the original researcher must bear full responsibility for the ethical aspects of the research. . . . The original researchers must take into account every single side of their research [including data sharing]. (Researcher–Male-I-27)

Many other researchers agreed that respect is an important cultural pillar that must be demonstrated by restricting duplication of existing studies, avoiding incongruity with the primary study results and ensuring that primary researchers have oversight of all potential ethical issues. It was also made clear that the data provider has a professional responsibility to liaise with the data user to ensure that the dataset is fully understood and that misinterpretation is avoided. Other factors commonly associated with increased acceptance of data sharing were as follows: having an existing relationship with the secondary data user, primary research being complete and published, increased age of the dataset and the perceived decline in utility, and limiting the amount of data shared as a proportion of the total data collected. A number of researchers and ethics committee members also stressed the obligation of the data user to contribute to building research capacity in Vietnam, though methods for doing so were not clear.

Consensus was strong among participants engaged in education, that sharing data with Vietnamese students who lack the resources to initiate independent data collection is important and has clear benefits:

In foreign countries, students are granted money to do research on their own but it is not the same in Vietnam. We should simplify the procedures to help students practice on real datasets. (Government officer–Male-I-13)

This highlights the common attitude that leveraging data sharing as a way to develop Vietnamese research is of core importance. Expanding access to data within Vietnam is thought to promote science nationally, delivering on the expectations of scientific benefit and capacity building with a local focus. Some researchers felt that sharing data outside of Vietnam would endanger the likelihood of achieving these local benefits, suggesting that when the data user is in another country, there is less assurance that any local benefit will be realized.

### Trust

Although not explicitly raised as a topic by participants, trust emerged as a theme throughout theoretical and practical discussions with all participants. The importance of personal and professional relationships in the culture of scientific research in Vietnam was highlighted by ubiquitous concerns regarding non-collaborative working practices. Trust in data quality, trust in secondary data users, and trust in researchers collecting data were each addressed from a variety of angles by respondents.

Data quality was the primary concern of many senior researchers and government officials. Many felt that the ability of a secondary data user to trust the data in a dataset would be compromised when direct involvement or link to data collection is absent. This strengthened the popular sentiment that data providers should have oversight of the outputs of data users’ prepublication. Others felt that increased quality standards in data collection, management, and tabulation should be met before datasets are shared:

I think data sharing is a good idea but data quality should be the first step. (Ethics committee member–Male-I-25)

Some participants extrapolated this risk to a potential reduction in the overall quality of science. It was felt that openly available data would discourage the likelihood of studies being replicated and promote uses of data that could be flawed:

I worry about another risk: if something has been done, obviously no one wants to do it again. Someone else did it, and then you just want to do other things. So there will be no one who cross-checks the results. This fact carries the risk of lack of verification [of shared datasets]. We only move forward but no one looks back for any potential gaps or mistakes in the past. (Researcher–Male-I-16)

The concept of relationships is a critical component of research collaboration in Vietnam. The trust built through collaboration is described as the “oil which lubricates the machine” of cooperation. Vietnamese researchers considered non-collaborative data-sharing models where the confidence that exists between known collaborators is absent. Without this link, there was concern that primary researchers could not prevent harms such as distortion of data or conflict of results with the original analysis:

What if the requestors figure out mistakes in the owners’ dataset? No one wants that (laughter). (Researcher–Male-I-18)

What if the requestors analyze and generate results different from the original owner? I have witnessed this scenario myself. They might use two different statistical methods, particularly foreign researchers pointing out mistakes in Vietnamese articles. (Researcher–Male-I-23)

Researchers generally did not accept the surrender of oversight of scientific and ethical standards to unknown data users. A number of individuals expressed trust in what were referred to as “prestigious institutions,” including the World Health Organization, and considered them more acceptable as data users, but when it came to sharing data with an anonymous individual, there was hesitation. Concern was also raised regarding the potential economic impact and commercial exploitation. Multiple participants referred to the use of Indonesian H5N1 strains for vaccine development during the 2000s:

You know, Indonesia got H5N1, they didn’t want to give data to the international community. They argued that they [the international community] do research to produce vaccines, to gain big profit by selling products at an expensive price while my patients are dying. . . . I think they are somewhat reasonable: it seems unfair. This story is very sensitive and subtle. If they keep going that way, there will be a time when cooperating stops! Unfortunately, such stories are still so common. . . . Sharing should have contributions from both sides. (Ethics committee member–Male-I-15)

There was consensus among ethics committee members that a contract between the data provider and data user, to define the terms of data use, could help to overcome concerns regarding unknown collaborators. Potential areas to be addressed in such contracts are outlined below in the Best Practices section.

Among patient representatives, there was a high level of confidence in the decisions of the research doctors. Consensus that data could be shared at the discretion of the researchers was unwavering. There was little concern for the future of the information. When potential personal risks were discussed, including exploitation, stigmatization, and loss of confidentiality, management of these risks was voluntarily given to researchers. The assignment of trust was independent of the nationality of the researcher. It was unclear whether this reflects confidence in international science, the research governance provided by the Vietnamese government, the strong involvement of Vietnamese doctors in all research, or other reasons. Representatives did not consider their input on the use of data to be a priority. A patient representative summed this up in a statement typical to the range of discussions on the topic:

No, I don’t have any concerns! You are a research institution; you should inform the region about their health problems to help them. We don’t have any concerns. You can give feedback to us as individuals if it helps us in some way to promote benefits or to prevent harms. That’s all! I see no reason to hide information! The more public it is, the better! Even [data on] diseases with the possibility of stigmatization and discrimination should also be public! (Patient representative–Male-G-01-05)

In contrast to the researchers, patient representatives did not consider the use of data for commercial profit to be a threat. After considering the risks and benefits of contributing to commercial research, one representative clarified the acceptance of data sharing for commercial purposes without direct benefit by stating,

We should not claim tangible benefits from pharmaceutical companies because in doing so we push drug prices higher. (Patient representative–Female-G-02-01)

The only concern identified by patient representatives was media and profiteers misusing patient data for the vested interests of companies. Although there was no objection to profits, there was disdain for current trends in the misuse or misrepresentation of facts to embellish marketing campaigns:

I am concerned about the possibility . . . that people might unreasonably trust a product’s quality. Media should be responsible for such misinterpretations. In foreign countries, there are medical staff in the newspaper to control the messages, but we still can’t do the same thing in Vietnam. (Patient representative–Female-G-02-02)

Sharing my information to researchers is good but don’t share it with the media. (Patient representative–Male-G-02-3)

Media or those who interpret the implications of the data in the media, should be qualified and make sure the interpretation is accurate. (Patient representative–Female-G-02-01)

### Expectations and Best Practice

In the interest of determining best practices for data sharing in Vietnam, the key issues of consent, reciprocity, and authorship are explored below.

### Consent

Opinions about the need to obtain consent for sharing data, and how much information is considered sufficient to make an informed decision, focused on practicalities and feasibility more than ethics. It was broadly agreed by all participants that to share data that have already been collected for a completed study, the responsible Ethics Committee could make a decision on behalf of the study participants. Reasons given to support this included the impracticality and resource requirements of tracing and contacting participants. There was agreement among patient representatives and researchers that the risk of disrupting someone’s day with an unnecessary phone call, or accidentally breaching the privacy of a patient by re-contacting him or her to give additional consent for data sharing, was greater than the potential risks in sharing the data. Regarding contacting patients to obtain additional consent, a patient representative noted,

I think the action of trying to establish individual identities to re-contact is ethically problematic in itself. (Patient representative–Female-G-02-01)

As well as informing the consent process, this consensus may demonstrate that data sharing is perceived as a relatively low-risk activity.

In considering what information should be given prospectively to study participants, there was heterogeneity in responses from research professionals. Responses spread evenly across a spectrum ranging from the requirement of explicit details of data-sharing plans to providing minimal information about future uses of data. In the opinion of one ethics committee member, “Broad consent is no consent at all” (Ethics committee member–Male-I-07). In contrast, a researcher took a different view:

There is no need to obtain another consent from patients for secondary [data] use except, perhaps for rare diseases only. And even if so, we should explain very generally because there more we explain, the more nervous the patients will be. (Researcher–Female-I-10)

Patient representatives were familiar with the principle and practice of obtaining informed consent. When asked if data sharing should be discussed in the informed consent process or if patients should be consulted regarding data sharing, there was strong consensus that this was unnecessary. In line with many responses regarding governance, representatives empowered researchers with the responsibility to make decisions on their behalf. This typical sentiment came from one representative:

We trust you [researchers]. You can use my information for different purposes. How should I know? We trust you! There is no reason to prevent data sharing. (Patient representative–Male-G-01-01)

When pressed to consider that consent was a standard requirement of conducting research, another patient representative suggested,

Perhaps you can explain in the consent form: “your information is only used for scientific development to promote health.” I would like to do that. According to this statement, other researchers can access my data to do further research. (Patient representative–Female-G-01-02)

### Reciprocity

When asked how to ensure that data sharing was implemented in a favorable and sustainable way in Vietnam, participating researchers proposed reciprocal models that supported capacity building and academic recognition for the data provider. As discussed above, benefiting scientific knowledge (ideally with relevance to Vietnam) was considered to be a core element of acceptable data sharing. This suggests that capacity building as a part of a data-sharing arrangement is seen as an important requirement for data sharing to be seen as acceptable. Indeed, capacity strengthening was explicitly stated to be a basic requirement by many:

At least, we expect investment in capacity building for us [Vietnamese researchers]. It is a very important point. (Researcher–Male-I-11)

The provision of resources was also seen as important to support data sharing locally. As doctors often undertake research as a task in addition to their regular job, it was considered important that compensation for the resources needed to share data was provided either by the funder or the data user. A researcher summed up the effect of the resource inequality between those driving data-sharing policies and Vietnamese researchers by explaining,

Scientific work in Vietnam and foreign countries is different. Vietnamese contribute great effort to do science, while personal financial gain and prestige are generally meager in comparison to developed countries. Their [Foreigners] salary is high and salary is their main source of income. Living on a meager salary, [Vietnamese] staff sometimes work tirelessly to generate a database. In the future, if salaries are in accordance to Vietnamese scientists’ contribution, I think the interest in data sharing will increase. (Researcher–Male-I-11)

Academic recognition was highlighted as critical by most senior Vietnamese researchers. This was proposed to be in the form of authorship on any papers resulting from secondary use of data. Researchers were also happy to be invited as collaborators, and subsequently authors, by data users. Some respondents said that it was important to ensure that those who collected the data are given appropriate credit for their efforts. Others stated that securing authorship was a means by which they could fulfill their responsibility as investigators to their patients by controlling the ethical and scientific standards applied to secondary data use:

Being an author is synonymous with holding ethical and scientific responsibility. Therefore, the secondary [data] user is the first author, then name of the owner of the original data stands next to it. (Researcher–Male-I-27)

When current international authorship guidelines were discussed with researchers and ethics committee members, it was accepted that requiring authorship as a condition of data sharing does not fit with international publication guidelines. Consequently, participants suggested that if the data provider is not an author, a review of the paper by the data provider should be required before publication. This concept contrasted with the views of most foreign researchers and some junior Vietnamese researchers interviewed. There was consensus among these groups that authorship should not be assigned on the basis of data provision:

Data comes from the patients, not from the investigators. So if others have some good ideas [about how to use data], they should use it. It is not my topic, so no, no . . . I don’t want to be the author! Maybe you’ve to mention where the data came from and the sponsors [in the acknowledgement section]. (Researcher–Male-I-26)

### Governance

Under the governance structure of the Vietnamese health care system, decisions regarding clinical research are delegated to the institutional scientific and ethics committees. There was agreement among all participating researchers, government officers, and ethics committee members that decisions regarding data sharing would also fall to these committees. All stakeholders shared the view that approval should be contingent on mitigating the risks of sharing data seen to be of increased sensitivity. Categories of potentially sensitive data included genetic and genomic data, and data about mental health and rare diseases. Community-level data that could lead to the stigmatization of specific ethnicities or were related to infectious disease outbreaks were also found to need increased governance. Rigorous de-identification was thought adequate to protect individuals, but oversight of the purposes for which data are used was considered important when results could have implications for communities or Vietnam:

. . . data implying differences among countries in term of lifestyle or such things, is sensitive. For example, when analysis points out that there is a difference between Thailand and Vietnam in terms of lifestyle, habit . . . which informs external people’s point of view, I consider it sensitive. It reminds me of a multi-site research on children who have experienced sexual abuse. If it is published, it will identify differences among Vietnam, Cambodia and Thailand. Then it is sensitive. That’s it. Otherwise, proportion, rate, number of cases . . . among countries, is the responsibility of media to publish. Analysis of such data is not of concern. (Researcher–Male-I-06)

## Discussion

The study results above examine the experiences, understandings, attitudes, and perceptions of data sharing among selected stakeholders in Vietnamese clinical research. The results delineate the expectations of what would constitute good data-sharing practice in Vietnam. In this “Discussion” section, we build on these findings to address how to support the development of culturally appropriate and locally acceptable data-sharing practice in Vietnam.

A significant finding of this study is the high level of trust placed in researchers by patient representatives. Patient representatives expressed willingness to entrust researchers with all decisions regarding the use of their data. They showed a low level of interest and lack of concern for personal risk with respect to data sharing. This contrasts with more mixed findings from Western countries where privacy, confidentiality, and distrust of for-profit institutions are more common (Lemke, Wolf, Hebert-Beirne, & Smitha, 2010; Trinidad et al., 2010). Participants in this study also showed a high level of acceptance of broad consent for data sharing and rejected the need or desire to be contacted for re-consent to share data for new purposes. These attitudes may be attributed to the culture in which the patient–doctor relationship is hierarchical and the doctor’s role is highly regarded, as described in a well-known Vietnamese saying “*Luong y nhu tu mau*” [“Doctors are gentle mothers”] (Donnelly, 2006).

This high level of trust grounds the rationale for Vietnamese researchers’ sense of ethical and professional responsibility toward their patient and thus, the need for oversight of the ethics of all future uses of data. From this point of view, Vietnamese researchers collectively assert that authorship should be shared with the data provider to ensure that the duty of the investigator who “stands in front of the patients,” to protect the scientific and ethical integrity of the data, is achieved. Although the sharing of authorship with data providers has been supported elsewhere (Vickers, 2006) as a means to prevent misinterpretation of data, this claim is incongruous with international guidelines. According to the International Committee of Medical Journal Editors’ “Defining the Role of Authors and Contributors,” authors must meet four criteria. “Substantial contribution . . . to data acquisition” fulfills only one of these criteria. These recommendations state that “Contributors who meet fewer than all four of the . . . criteria for authorship should not be listed as authors, but they should be acknowledged” (International Committee of Medical Journal Editors, 2013). This has been directly addressed in the context of data sharing by others who concluded that authorship is not appropriate when sharing of data is a researcher’s only contribution to a particular article (Rohlfing & Poline, 2012). Co-authorship is additionally important to the academic recognition systems within Vietnam and can build trust with data providers. However, this approach may prompt concerns of selective reporting as it excludes the benefit of accountability when the data provider can prevent the publication of papers that corrects errors in the original research (Rising, Bacchetti, & Bero, 2008; Smith & Roberts, 2006). These are important considerations for funders, publishers, and others imposing data-sharing requirements to promote equity and ensure the returns sought by lower income data providers.

The altruistic prioritization of community benefit over personal interests is also evident in the views of the patient representatives. The significance assigned to community benefit over individual benefit mirrors the trend described in ethics guidelines of other resource-limited settings (Lairumbi, Parker, Fitzpatrick, & English, 2011). As existing guidelines and policies on data sharing are mostly driven by well-resourced Western institutions and publishers, it is important to ensure that the development of data-sharing practice in Vietnam takes into account the priorities of the study participants who provide the data.

The findings of this qualitative study provide insights into the challenges related to sharing clinical and public health research data collected in Vietnam. The data collected represent the experience of a small number of government officers, ethics committee members, researchers, and research participants and are therefore not meant to characterize any specific population. However, individuals from a range of locations, socio-economic backgrounds, and levels of experience with data sharing were included in the population, and consistency in the findings suggests that the ideas presented reflect many of the key issues regarding data sharing in the Vietnamese context. Additional study limitations include the challenges of elucidating participants’ perspectives about a novel and unfamiliar topic. Background and introductory discussions with the study staff may have affected participants’ initial opinions of data sharing. Furthermore, discussion of perspectives about data sharing in the absence of a regulatory framework was met with a cautious response, as it is not customary for stakeholders to discuss issues that fall outside of their assigned responsibilities.

## Best Practices

One of the outcomes of this study is a framework document, drafted by the lead authors and revised during a series of workshops with ethics committee members and researchers in Ho Chi Minh City between December 2014 and June 2015. The final document is expected to be published by the end of 2015. This exercise was a practical way to define the foundation of best practice informed by the major research institutes in the region. The resulting framework is now a tool to initiate ideas and discussion among the committees responsible for governing data sharing and should continue to evolve. The document is characterized by a culture of reciprocity, relying on professional trust rather than legal guarantees. This is consistent with views of data sharing in other developing settings (Tangcharoensathien, Boonperm, & Jongudomsuk, 2010). According to the contributors to this framework document, key principles of data sharing in Vietnam that should underpin governance and policy are

- to ensure that the rights and interests of research participants and their community are safeguarded, including preserving privacy, the right to dignity, protection from harm, and appropriate sharing of benefits;
- to protect rights and interests of primary researchers, particularly given the potential inequalities in resources available to support local analysis and publishing; and
- to be transparent and accountable.

High levels of trust in researchers were found among patient representatives. Thus, it is understandable that broad consent was accepted among the Vietnamese study population, who rejected the need or desire to be contacted for re-consent for new uses of data. However, despite this inherent trust, the autonomy of participants should be respected and reinforced. In addition, the range of opinions among researchers and ethics committee members about appropriate forms of consent may relate to the novelty of the topic and is worthy of further exploration as data-sharing initiatives evolve across Vietnam.

In line with the International Ethical Guidelines for Biomedical Research Involving Human Subjects (The Council for International Organizations of Medical Sciences, 2002) and the work of others (Mascalzoni, Hicks, Pramstaller, & Wjst, 2008; Murphy et al., 2009), we believe that a participant’s autonomy should be supported through the provision of information regarding all potential uses of data during a robust informed consent process, potentially supplemented by feedback about secondary uses of data. Providing participants with an option to receive further information on the original consent form may decrease unwanted feedback. In addition, public engagement activities about data sharing should extend beyond the individual to include community engagement.

Compliance with best practices in data sharing is dependent on available financial and technical resources. This is especially important in low- and middle-income settings (Pisani & AbouZahr, 2010), and Vietnam is no exception. To promote local engagement in the development of best practices, we recommend engagement with the government and policy makers to prioritize and formalize such initiatives.

## Research Agenda

This study has produced a number of novel findings about data sharing in Vietnam, which deserve further exploration to identify appropriate policy responses. Determining the best way to obtain informed consent for data sharing and how to inform the community of developing ideas around the topic should be prioritized. As policy is centrally developed in Vietnam, relevant government actors should be consulted to determine what information will best support the current priorities. Additionally, as data sharing is introduced to Vietnam, it is important to track its uptake, challenges, and evolution of practices to determine how to support such sharing and ensure that the priorities and returns to the Vietnamese community are protected.

## Educational Implications

Researchers participating in this study were found to have a number of reservations about sharing the data collected in their own studies. We argue that these reservations are motivated by concerns that can be addressed by appropriate data-sharing policies and practices. First, concerns about the sensitivity of sharing data were attributable to the absence of explicit government policy. Within a health care system that relies heavily on legal framework, a lack of official policy can stagnate action. Adding to this concern is a lack of awareness of established data-sharing guidelines on de-identification and preparation of data for sharing. Second, reservations were found to arise from misconceptions regarding data ownership. Beliefs that all data belong to the research funders and that research data are protected and copyrightable by intellectual property law (The Association of Learned & Professional Society Publishers [ALPSP], 2014) were common. Third, misgivings about competitive advantage were partly attributable to the lack of familiarity with the novel concept of data sharing and its potential benefits. These findings suggest that there is opportunity to facilitate the uptake of locally appropriate data-sharing practice in Vietnam through the provision of training and resources. When the knowledge gaps are addressed, we believe that the development of ideas around culturally appropriate uptake will be promoted.

The findings of this study additionally highlight the need to inform the developers of international data-sharing policies about the priorities of stakeholders in low- and middle-income countries. International funders and publishers should consider the results of this and further studies to ensure that the interests of all research communities are safeguarded by the policies they develop.

## Supplementary Material

Supplementary material

## Supplementary Material

Supplementary material

## Supplementary Material

Supplementary material

## Supplementary Material

Supplementary material
