# Supplementary material for: Trust, Respect, and Reciprocity: Informing Culturally Appropriate Data-Sharing Practice in Vietnam
Source: J Empir Res Hum Res Ethics. 2015 Jul;10(3):251–63. doi: 10.1177/1556264615592387 (PMC4692260; doi:10.1177/1556264615592387)
Supplement: Supplementary material [file 02EP_Interview_guide_for_IRB_members_and_health_managers_592387.pdf]

INTERVIEW GUIDE FOR IRB MEMBERS AND HEALTH MANAGERS

Study title: Examining the views of stakeholders in Vietnam on how public health research data should be shared

**Demographic Information**	
1. Full name:	2. Interviewee identifier: 02EP-[___][___]-[___][___]
3. Age:	4. Gender: ○Male ○Female
5. Ethnicity:	6. Religion:
7. Nationality:	8. Employer:
9. Place of interview:	10. Interview date: [__|__]/[__|__]/[__|__]<br>Date Month Year
11. Time start and end: _________to____________	12. Interviewed by: ________
13. Note taker:________	14. Voice recorded? [Y] / [N]

Questions:

BACKGROUND

1) Can you briefly describe your professional background?
2) Can you briefly describe the role and responsibility of the ethics committee/organization in research?
3) What is your role in your committee/organization? How long have you been in this?

EXPERIENCE IN RESEARCH ETHICS REVIEW AND MANAGEMENT

4) Can you describe your experience in reviewing, managing and overseeing the research in terms of difficulties, challenges, ethical issues/consideration and advantages?
5) Why did your committee/organization see them as difficulties/challenges/ethical considerations/issues/advantages?
6) How did the committee/organization resolve the problems? If nothing could be done, why not?
7) Did you find the solutions satisfied? If not, do you have any other suggestion to improve them?

OWN EXPERIENCE

8) In any research you've been involved in reviewing, managing and overseeing, have you been involved in/come across the concept of researchers sharing the information they either collect or find out during studies with other local/international researchers/organizations? Can you tell me about this situation/these situations? What type of research/data? What was the purpose of sharing? Were you a data sharing reviewer/policy maker? What was the

process of reviewing/policy making? Who made decisions about whether the data could be shared and how?

9) What was your view about sharing data in this situation? What were the reasons you felt this? *Probe for perceptions and experiences of issues for & against sharing data in this situation, and reasons for these views. After listening to views, probe on ethical issues not raised directly, including participant/community confidentiality, autonomy, benefit sharing; primary researchers' interests; authority; governance.*

10) What kind of data policies or guidelines, including data sharing policies/guidelines relating to this situation did you/your organization operated according to? Were they the national guidelines or your own policies? Was there any case that you/your organization apply both? What did you think about the advantages/disadvantages and why?

11) Which are the important points in the policy development process? Who are your stakeholders in the policy development process? What is the process of data sharing? What are the forms of institutional accountability?

12) What do you think about the best way for decisions to be taken about requests from other researchers for access to data in this database? Who should be involved in making these decisions? Which policies should be operated to? Do you have any suggestion that the policies should be amended or changed? *Probe for views on appropriate governance mechanisms for handling requests for data sharing.*

13) *[If no experience of data sharing*] Have you heard anything about the idea that researchers should try to share data where they can with other local/international researchers/organizations? Can you tell me what you think in general about this idea? *Probe for knowledge about data sharing concepts; main issues seen for & against sharing data in any situation they raise; and views about which types of data have most utility & challenges in relation to sharing, and why.*

    [If have never heard of concept of data sharing, outline key issues here including: scientific utility; participant/ community confidentiality, autonomy, sharing benefits; primary researchers' interests; authority; governance and other relevant ethical issues. Use examples to illustrate].

SCENARIO - SHARING DATA ON INFECTIOUS DISEASE OUTBREAKS – ISARIC

Explain the background to the ISARIC-WHO project on Acute Respiratory and Emerging Infections and the currently legal documents issued by Vietnam government and Ministry of Health

14) What is your view about sharing data in this situation? What should be shared or not be shared? What types of purposes can be acceptable, unacceptable? What are the reasons you feel this and why? *Probe for perceptions and experiences of issues for & against sharing data in this situation, and reasons for these views. After listening to views, probe on ethical issues not raised directly, including participant/community confidentiality, autonomy, benefit sharing; primary researchers' interests; authority; governance & trust; economic effect.*

15) There are currently some legal documents issued by Vietnam government and Ministry of Health, detailing

different aspects of communicable disease prevention and control, including the acute respiratory and emerging infections. In your opinion, which articles in the legal documents can be applied for this situation? What kind of other data sharing policies, including data sharing policies relating to this situation do you think you should operate according to? What do you think about the advantages/disadvantages relating to operating these policies and why? If there is any disadvantage/problem, do you have any suggestion to solve it?

16) What do you think about the importance of sharing data with i) the government/Ministry of health/other researchers from Vietnamese public health organizations; ii) other researchers in other similar programs within Vietnam; iii) other researchers in other programs in Vietnam; iv) other researchers in the ISARIC programs outside Vietnam; v) other researchers outside Vietnam for any new research; vi) other international public health researchers/organizations for any new research; vii) pharmaceutical companies/those paying for the data? What do you think about any ethical challenges this might involve? *Probe for perceptions and experiences of issues for & against sharing data in this situation, and reasons for these views.*
17) Can you tell me about your views about the different purposes of sharing data in this situation? *Probe for views about patient/community member care/benefit, national health priority, academic/medical/commercial purpose, unknown future use…*
18) What do you think about the rights and obligations of sponsors, researchers, recipients, participants and other stakeholders (if yes) in this situation? *Probe for patents, publication, storage/manage/control, decision for additional use, abuse of data and other relevant issues.*
19) What would be a useful resource for the recipients? *Probe for length, format of storage, future availability, releasing time.*
20) What do you think about the best way for decisions to be taken about requests from other international researchers/organizations for access to data in this database? Who should be involved in making these decisions? *Probe for views on appropriate governance mechanisms for handling requests for data sharing.*
21) In your opinion, what would be the best data sharing guidelines/policies that can meet the request of sharing data from the community of researchers, concurrently ensure the rights and the benefits of the researchers and the participants?

General comments on interview:
