# Supplementary material for: Trust, Respect, and Reciprocity: Informing Culturally Appropriate Data-Sharing Practice in Vietnam
Source: J Empir Res Hum Res Ethics. 2015 Jul;10(3):251–63. doi: 10.1177/1556264615592387 (PMC4692260; doi:10.1177/1556264615592387)
Supplement: Supplementary material [file 02EP_Interview_guide_for_Patients_and_Their_relatives_592387.pdf]

INTERVIEW GUIDE FOR PATIENTS AND THEIR RELATIVES

Study title: Examining the views of stakeholders in Vietnam on how public health research data should be shared

**Demographic Information**	
1. Full name:	2. Interviewee identifier: 02EP-[___][___]-[___][___]
3. Age:	4. Gender: ○Male ○Female
5. Ethnicity:	6. Religion:
7. Nationality:	8. Employer:
9. Place of interview:	10. Interview date: [__|__]/[__|__]/[__|__]<br>Date Month Year
11. Time start and end: _________to____________	12. Interviewed by: ________
13. Note taker:________	14. Voice recorded? [Y] / [N]

Questions:

General information about the research

1) Which research projects have you/your relatives been involved in?
2) Can you briefly tell me what you know about the research that you/your relatives have participated?
3) What made you decide to participate in the research?
4) Have you/your relatives participated in other research? Can you briefly tell me what that research is about?

Experience in research participation

5) Have you had any experience of you/your relatives known being asked to participate in a study?
6) In what way did the study staff approach you/your relatives and ask for your information during the time you/they were asked for consent/at the out-patient ward/hospitalized? What information did they collect? What did you/they think about this?
7) For any information you/they gave, and any information from medical examinations and tests, what do you think about what happens to the information researchers collect from participants or generate in this study? *Probe for uses within the program and any other, including hopes and concerns about possible uses in program or outside. Look particularly for any ideas about transfer of data outside the program, and explore how, where to and views about this. At end of this discussion, summarize types of information typically collected and why.*
8) What do you feel about giving this information and why? What about others in your household? *Probe for types &*

perceptions about benefits and concerns/issues with either purpose or organizations.

9) Did you know what would happen to your/their data? How did you/they find out how the information would be used? Did you/they know who would use your information? And what did you think about this? *Probe for information given by study staff recruiting the participants as a part of consent process.*

SCENARIO- SHARING DATA ON INFECTIOUS DISEASE OUTBREAKS

Describe ISARIC study: *Building on the information provided above, using non-technical language to summarize: i) the aims of this study, ii) the aims of data collection, iii) the types of data collected directly from participants and generated indirectly by primary researchers in the study, iv) how consent given by participants; v) how stored including pseudo-anonymization of data; vi) aiming to share data between different countries to support research towards improvement of health*

10) Since ISARIC data/information can be used in many ways to support health planning and research in the future, researchers cannot know all these possible future uses at the time the information is collected. *Remembering that all data are anonymised,* what do you think if other local researchers/organizations request to access this data for their own purposes? *Use examples if necessary.*

11) Do you think that researchers of this study should agree to give other local researchers/organizations access to information they have asked for? Why and why not? How would you compare these two sets of issues? Overall, what should researchers do? *Probe for perceptions of potential harms/wrongs for people who gave information, or for wider community/others, and issues of confidentiality, including to authorship, sharing benefits or others. Probe for perceptions of importance of sharing information, including value of research to other researchers, to the community as well as other communities that the researchers might serve in future.*

    [If think ok to share] Are there any ways you would want requests like this to be checked before being approved? Who should decide this?

    [If see more risks of harms/wrongs in this situation] Are there any ways in which researchers could address this problem so that the data could be shared? What is this? How do you think researchers do this at the moment?

12) What if the researchers asking for this data came from another country? Would this make a difference to your view on data sharing? How and why?

    [If think ok to share] Are there any ways you would want requests like this to be checked before being approved? Who should decide this?

    [If see more risks of harms/wrongs in this situation] Are there any ways in which researchers could address this problem so that the data could be shared? What is this? How do you think researchers do this at the moment?

13) What if researchers were asked to share data with a wide network of international researchers working on public health issues, through putting up information on rates of birth/death or anonymized individual data on these on a

public website?

[If think ok to share] Are there any ways you would want requests like this to be checked before being approved? Who should decide this?

[If see more risks of harms/wrongs in this situation] Are there any ways in which researchers could address this problem so that the data could be shared? What is this? How do you think researchers do this at the moment?

14) In general, what kinds of data (of those collected during the ISARIC study) do you see as more sensitive than others? Why?

CONSENT & GOVERNANCE PROCESSES

Explain that nowadays many research institutions are increasingly expected to share data as much as possible in order to maximize utility of data to find better ways of preventing and treating illness in the future for everybody's benefit. Many see that it is wrong for data collected by researchers not to be used as much as possible so that the participants' contributions are properly recognized and that participants are not asked to participate in studies unnecessarily. In some studies, at the time people are asked if they will give information, it is not possible to know exactly how the data might be used in the future. This means that researchers cannot explain to people who give information how that information will be used. There is therefore a possibility that information could be used in ways that people are not aware of and/or might not have agreed to. There are 2 possible ways that researchers have thought about trying to deal with this issue. These are:

- *Asking people to agree to their anonymised data being used for any purpose in future, but with a specially planned committee to look at requests and decide is they are reasonable.*
- *Asking people to agree to their anonymised data being used in future, but making sure that those who give information can explain any limits to the way they would like to see the information used.*

15) What do you think about the way researchers should ask you for consent for use of the information collected? Does it limit the way data can be used in future? Why/why not?
16) In this research, we are asking for your permission to share the interview/focused group discussion data (transcripts) with other researchers conducting the similar topics. Can you describe the reasons why you would or would not agree to share?
17) In case you agree/disagree to share the data, including the interview data, but your relatives have the opposite opinions, what do you think the researcher should do?
18) In case you agree to share the data collected from your under-18-year-old child/relative participate in the study, including the interview data, then he/she comes of age (≥ 18 years old) and does not agree to share his/her data, what do you think the researchers should do?
19) In other places, it has been suggested that researchers should: i) come back to ask you and/or your relatives

about every new request for use of this data; ii) during the consent process, ask for your and/or their permission to use the data for ‘any purpose’ in future and ***have special committee/s to check each request on participants’ behalf***; iii) during the consent process, ask for consent to use the information for limited purposes and come back to you and/or them for purposes outside these uses. Which would you prefer (or neither), and why? Is there any better way?

20) If option ii) preferred, who would you feel confident about/trust to make decisions about data sharing requests on your behalf and/or your relatives’ behalf? *Probe for types of individuals who should be involved, and reasons for this, including: researchers/Wellcome Trust/local and national ethical committees/health managers?*
21) If option iii) preferred, what kinds of limits would you want to put on future use before you and/or your relatives should be asked for permission?
22) What do you think about sharing data with: i) the government/Ministry of health/other researchers from Vietnamese public health organizations; ii) other researchers in other similar programs within Vietnam; iii) other researchers in other programs in Vietnam; iv) other researchers in the ISARIC programs outside Vietnam; v) other researchers outside Vietnam for any new research; vi) other international public health researchers/organizations for any new research; vii) pharmaceutical companies/those paying for the data? Does it make any difference with whom this information is shared, and why does this matter to you?

General comments on interview:
