# Supplementary material for: Trust, Respect, and Reciprocity: Informing Culturally Appropriate Data-Sharing Practice in Vietnam
Source: J Empir Res Hum Res Ethics. 2015 Jul;10(3):251–63. doi: 10.1177/1556264615592387 (PMC4692260; doi:10.1177/1556264615592387)
Supplement: Supplementary material [file 02EP_Interview_guide_for_Research_and_Hospital_staff_592387.pdf]

# INTERVIEW GUIDE FOR RESEARCH STAFF AND HOSPITAL STAFF INVOLVING IN RESEARCH

## *__Study title:__* Examining the views of stakeholders in Vietnam on how public health research data should be shared

| **Demographic Information** | |
|---|---|
| 1. Full name: | 2. Interviewee identifier: 02EP-[___][___]-[___][___] |
| 3. Age: | 4. Gender: ○Male ○Female |
| 5. Ethnicity: | 6. Religion: |
| 7. Nationality: | 8. Employer: |
| 9. Place of interview: | 10. Interview date: [__\|__]/[__\|__]/[__\|__]<br>Date Month Year |
| 11. Time start and end: _________to_____________ | 12. Interviewed by: ________ |
| 13. Note taker:________ | 14. Voice recorded? [Y] / [N] |

**Questions:**

**GENERAL INFORMATION ABOUT RESEARCH AND RESEARCH WORK**

1. Have you ever been involved in research projects?
2. Have you ever been involved in severe acute respiratory infection research projects?
3. Have you worked full time for research or do you have another job while partaking in the research project? What work takes up most of your time?
4. Can you describe how participating in these research projects affect your work?
5. Does the research involve any collaboration? If yes, who are the local and/or international partners? And what role do they play in the research?
6. How many projects does your organization conduct per year? Who are the local and/or international partners in the projects?
7. Has there been any decision taken not to collaborate because of issues around research data sharing? Can you describe this situation?

**OWN EXPERIENCE**

8. In any research you've been involved in conducting, have you been involved in/come across the concept of researchers sharing the information they either collect or find out during studies with other researchers? Can you

tell me about this situation/these situations? What types of research/data/purposes? Were you a data user/data provider? What types of data did you produce, hold, use, seek to access? What was the process of data sharing? Who made decisions about whether the data could be shared and how? What did you think about this situation? *Probe for perceptions and experiences of issues for & against sharing data in this situation, and reasons for these views. After listening to views, probe on ethical issues not raised directly, including participant/community confidentiality, autonomy, benefit sharing; primary researchers' interests; authority; governance.*

9. Were there any researchers from other national or international organizations wanted to access the database for their own research purpose? What types of data should/should not be shared? What was a useful resource for them? What were the reasons you felt this?

10. Did you see yourself as a part of this data sharing decision-making process? What did you think about the best way for decisions to be taken about requests of this nature from other researchers for access to data in this database? Who should be involved in making these decisions? *Probe for views on appropriate governance mechanisms for handling requests for data sharing of this type.*

11. Have you ever requested to access the databases from other organizations? What types of data did you request to access? Why did you want to access the databases? Can you tell me about the advantages/disadvantages in this situation? What were the reasons you felt this? *Probe for perceptions and experiences of issues for and against in this situation, and reasons for these views.*

12. Can you describe the research policies of your institution? Does your institution develop any policy relating to data sharing? If yes, can you tell me about this? Which are the important points in the process? Who are your stakeholders in the policy development process? What role do the sponsors, researchers, recipients, participants and other stakeholders (if yes) play in your data sharing policy? What is the process of data sharing? What are the forms of institutional accountability?

13. What types of data policies do you think you have to operate according to? Are they the national policies or the own policy of your organization or both?

14. In what way do you think the nature of data sharing can facilitate your work? *Probe for the positive effects of sharing data on their works.*

15. *[If no experience of data sharing*] Have you heard anything about the idea that researchers should try to share data where they can with other researchers? Can you tell me what you think in general about this idea? *Probe for knowledge about data sharing concepts; main issues seen for & against sharing data in any situation they raise; and views about which types of data have most utility & challenges in relation to sharing, and why.*

*[If have never heard of concept of data sharing, outline key issues here including: participant/ community confidentiality, autonomy, benefit sharing; primary researchers' interests; authority; governance and other*

*relevant ethical issues. Use examples to illustrate]*

**SCENARIO - SHARING DATA ON INFECTIOUS DISEASE OUTBREAKS – ISARIC**

**Explain the background to the ISARIC-WHO project on Acute Respiratory and Emerging Infections**

16. What is your view about sharing data in this situation? What types of data can be shared? What types of sharing purpose can be acceptable, unacceptable? What is the reason you feel this? *Probe for perceptions and experiences of issues for & against sharing data in this situation, and reasons for these views. After listening to views, probe on ethical issues not raised directly, including participant/community confidentiality, autonomy, benefit sharing; primary researchers' interests; authority; governance.*
17. What do you think about the importance of sharing this information with i) the government/Ministry of health/other researchers from public health organizations; ii) other researchers in other similar programs within Vietnam; iii) other researchers in other programs in Vietnam; iv) other researchers in the ISARIC programs outside Vietnam; v) other researchers outside Vietnam for any new research; vi) other international public health researchers/organizations for any new research; vii) pharmaceutical companies/those paying for the data? What do you think about any ethical challenges this might involve? *Probe for perceptions and experiences of issues for & against sharing data in this situation, and reasons for these views.*
18. What do you think about the roles, rights and obligations of the sponsors, researchers, recipients, participants and other stakeholders (if yes) in data sharing?
19. What do you think about the advantages/disadvantages you might encounter when you obtain participants' consent for sharing data? If there is any problem, do you have suggestion to solve it?
20. What do you think about the best way for decisions to be taken about requests from other researchers for access to data in this database? Who should be involved in making these decisions? *Probe for views on appropriate governance mechanisms for handling requests for data sharing.*
21. What can be a useful resource for researchers who request to access the database? How to maximize utility of shared data?
22. In your opinion, what would be the best data sharing guidelines/policies that can meet the request of sharing data from the community of researchers, concurrently ensure the rights and the benefits of the researchers and the participants?

*General comments on interview:*
