# Supplementary material for: Trust, Respect, and Reciprocity: Informing Culturally Appropriate Data-Sharing Practice in Vietnam
Source: J Empir Res Hum Res Ethics. 2015 Jul;10(3):251–63. doi: 10.1177/1556264615592387 (PMC4692260; doi:10.1177/1556264615592387)
Supplement: Supplementary material [file Informed_Consent_Form_592387.pdf]

# Informed Consent Form

## Study title: Examining the views of stakeholders in Vietnam on how public health research data should be shared

**What is this study about?**

Hospital for Tropical Diseases, Children's Hospital No.1, HCMC and the Oxford University Clinical Research Unit (OUCRU-VN) are collaborating on a study to learn more about what Vietnamese doctors, patients and community members think about sharing health research data. The study will focus on understanding the opinions, concerns and hopes about what will happen to research data and how researchers in Viet Nam protect and share health research information in line with the wishes of the Vietnamese community. The findings from this study will be used to inform ethical policies on how to share data. You are invited to take part in this study because your opinion about research data sharing is important to us. Your decision to take part is completely voluntary.

**What will happen if you take part?**

If you agree, you will take part in 1-2 individual interviews/group discussions of up to 90 minutes for individual interview and up to 120 minutes for group discussion to discuss your understandings, experiences and concerns about data sharing and what might constitute good data sharing practice. The discussions may be between you and the interviewer, or with a number of other people with similar experience/knowledge of data sharing. If you do not want to answer any of the questions you may say so and the interviewer will move on to the next question. The researchers would like to record and transcribe the interview(s)/group discussions for analysis. The interviews/discussion(s) will take place at Hospital for Tropical Diseases, Children's Hospital No.1, HCMC and OUCRU.

**What will happen to the information collected?**

No one else but the interviewers or the people joining the discussions will be present there. The information from the interviews/discussions will be coded and stored. Any information that can identify you has been removed from your data. Only the researchers named in this protocol can access your data.
The ideas and opinions of everyone who takes part in the study will be shared anonymously with other researchers who are interested or contributing to the development of data sharing practice.

**Are there any disadvantages or benefits to me of taking part?**

We do not think that there will be any disadvantages of taking part, apart from the time taken in discussion. The discussions should take approximately 1-2 hours. In talking to us, you will contribute to developing policies on how research data (information) should be shared.

When you attend a discussion, you will be reimbursed VND 200,000, plus a small travel fee if you came to the Hospital or OUCRU especially for the discussion. The study staff can tell you what the travel fee is according to the distance from your home from the hospital/OUCRU.

**Do you have to take part?**

You are free to decide if you want to take part or not. If you do agree you can change your mind at any time without any consequences. You may also withdraw the data collected from the individual discussions at any time before the analysis begins. You will not be able to withdraw data that they have already contributed to group discussions as it can be very difficult in practice to ensure that all of an individual participant's comments have been withdrawn, and attempting to withdraw such data may compromise the integrity of the data that others have contributed.

**How can you get more information?**
Please discuss all of your questions with the study staff before deciding to participate. If you would like more information about this work, you can contact:

1. The Research Office at the Hospital for Tropical Diseases at {08 39241983}

2. Dr. Nguyen Thanh Dung – Hospital for Tropical Diseases, HCMC at {08. 39235476}

3. Dr. Le Nguyen Thanh Nhan – Children's Hospital No.1, HCMC at {08. 39274247}

If you would like a copy of the policy recommendations that arise from this project, please contact us at 389241983 or give us your email address.

**What do I do if I want to participate?**
When you have had an opportunity to read this form, discuss this study with a member of the research team and had your questions answered satisfactorily, please complete the section below. (Please check what you agree with and leave blank the ones that you don't agree with).

☐ I understand that my participation is voluntary, and that I am free to withdraw from the study at any time and it will not affect me in any way.

☐ I agree to take part in 1-2 interview(s)/discussion(s)

☐ I agree to the interview(s)/discussion(s) being recorded

☐ I agree for an anonymised written record of the interview(s)/ discussion(s) to be shared with other researchers studying similar topics.

By signing my name/making my mark here, I confirm what is written above.

| | | |
|---|---|---|
| x_________________________ | x_____________________ | ___/____/_____ |
| **Participant Signature** | Print Name | Date of Signature |

I, the undersigned, have fully explained the relevant information of this study to the person named above and will provide her/him with a copy of this signed and dated informed consent form.

| | | |
|---|---|---|
| x_____________________ | x________________ | ___/____/_____ |
| **Investigator/Designee Signature** | Print Name | Date of Signature |

**If the person giving consent cannot read the form themselves, a witness must be present and sign here:**
This form was read accurately to the volunteer, all questions from the volunteer were answered and the volunteer has agreed to take part in the research.

| | | |
|---|---|---|
| x________________ | x_______________________ | _____/______/_____ |
| **Witness Signature** | Print Name | Date of Signature |
